# *TERT* Promoter Mutation as a Potential Predictive Biomarker in BCG-Treated Bladder Cancer Patients

**DOI:** 10.3390/ijms21030947

**Published:** 2020-01-31

**Authors:** Rui Batista, Luís Lima, João Vinagre, Vasco Pinto, Joana Lyra, Valdemar Máximo, Lúcio Santos, Paula Soares

**Affiliations:** 1 Instituto de Investigação e Inovação em Saúde (i3S), 4200-135 Porto, Portugal; rbatista@ipatimup.pt (R.B.); jvinagre@ipatimup.pt (J.V.); vmaximo@ipatimup.pt (V.M.); 2 Instituto de Patologia e Imunologia Molecular da Universidade do Porto (IPATIMUP), 4200-135 Porto, Portugal; vasco.sa.pinto@gmail.com (V.P.); joanaritalyra@gmail.com (J.L.); 3 Faculdade de Medicina da Universidade do Porto (FMUP), 4200-319 Porto, Portugal; 4 Grupo de Patologia e Terapêutica Experimental, Instituto Português de Oncologia do Porto FG, EPE (IPO-Porto), 4200-072 Porto, Portugal; luis14lima@gmail.com (L.L.); llarasantos@gmail.com (L.S.)

**Keywords:** *TERT* promoter mutations, *FGFR3*, non muscle invasive bladder cancer, BCG therapy

## Abstract

Telomerase reverse transcriptase gene promoter (*TERTp*) mutations are recognized as one of the most frequent genetic events in bladder cancer (BC). No studies have focused on the relevance of TERTp mutations in the specific group of tumors treated with Bacillus Calmette–Guérin (BCG) intravesical therapy. Methods — 125 non muscle invasive BC treated with BCG therapy (BCG-NMIBC) were screened for *TERTp* mutations, *TERT* rs2853669 single nucleotide polymorphism, and Fibroblast Growth Factor Receptor 3 (*FGFR3*) hotspot mutations. Results — *TERTp* mutations were found in 56.0% of BCG-NMIBC and were not associated with tumor stage or grade. *FGFR3* mutations were found in 44.9% of the cases and were not associated with tumor stage or grade nor with *TERTp* mutations. The *TERT* rs2853669 single nucleotide polymorphism was associated with tumors of higher grade. The specific c.1-146G>A *TERTp* mutation was an independent predictor of nonrecurrence after BCG therapy (hazard ratio—0.382; 95% confidence interval—0.150–0.971, *p* = 0.048). Conclusions — *TERTp* mutations are frequent in BCG-NMIBC and -146G>A appears to be an independent predictive marker of response to BCG treatment with an impact in recurrence-free survival.

## 1. Introduction

Bladder cancer (BC) ranks as the fifth most common cancer in western society and the sixth most prevalent in the world, with an increasing incidence in the past years [1]. The increased incidence, along with the high costs in surveillance per BC patient, results in a high burden for public health systems [2,3]. BC can be divided in non muscle invasive (NMI) and muscle invasive (MI) tumors. NMI bladder cancer (NMIBC) accounts for 70% to 80% of all BC and is present as superficial and recurrent lesions that only seldom progress to an MI phenotype. Prompt treatment, usually with complete transurethral tumor resection, grants a 5-year survival rate that can surpass 90%. However, up to 70%-80% of them may relapse, making recurrence the main challenge in clinical management [3,4]. Present in approximately 70% of cases, Fibroblast Growth Factor Receptor 3 (FGFR3) activating mutations are the most frequent genetic event in the NMI phenotype [4]. MI bladder cancer (MIBC) accounts for the remaining 20% to 30% of BC cases and presents as an invasive tumor at diagnosis. Characterized by a high risk of distant metastasis, MIBC prognosis is considerably worse, with 5-year survival rates often described as lower than 40% [5]. MI tumors are genetically more heterogeneous than NMI tumours; present in approximately half of the cases, *TP53* mutations are identified as the most frequent genetic alteration in these tumors [4].

Cell immortalization is a classic hallmark of cancer cells and telomerase reactivation is proposed to be involved in the underlying process. In a large part of cancer models, the intervening mechanisms remained elusive, until in 2013, mutations of the promoter of the telomerase (*TERT*) were described in melanoma [6,7]. We and others reported for the first time the presence of recurrent somatic mutations in the *TERT* promoter (*TERTp*) in numerous types of cancer, including BC [8,9,10,11,12,13,14,15]. Studies focusing on BC have described a prevalence of *TERTp* mutations ranging from 52% to 85% of the cases [10,13,14,16,17,18,19]. Conflicting results have been obtained on the association between *TERTp* mutations and BC clinical outcome [13,14]. A common polymorphism in *TERTp* (rs2853669 single nucleotide polymorphism) is also accountable to act as a modifier of the promoter mutations’ effect on survival and tumor recurrence in several cancers, such as glioblastoma, liver, and bladder cancer [18,20,21].

Clinicopathological features are the central determinants of recurrence, and according to the European Organization for Research and Treatment of Cancer (EORTC), the NMIBC high-risk group includes high-grade papillary tumors, carcinoma in situ, and those with multifocal or recurrent lesions [22]. Tumor resection followed by a schedule of intravesical instillations with Bacillus Calmette–Guérin (BCG) is the standard adjuvant therapy for this high-risk group (henceforth referred to as BCG-NMIBC) [22,23]. Nonetheless, 30% to 40% of patients present either intolerance or recurrence following BCG treatment, demanding a life-long follow-up and repeated courses of treatment [24]. This clinical relevance is recognized and there is a shortage of dedicated genetic markers predicting BCG-NMIBC subgroup outcomes, in particular, the now recognized two most common genetic events in NMIBC—*TERT* promoter and *FGFR3* mutations.

In this study, we screened a series comprising 125 BCG-NMIBCs resected before BCG therapy initiation for *TERTp* mutations, *FGFR3* mutations, and for the *TERTp* rs2853669 polymorphism. This represents a unique report of *TERTp* and *FGFR3* mutation genotyping dedicated to the BCG-NMI group of BC. To investigate the significance of *TERTp* mutations in the BCG-treated tumor response, we compared the obtained results with the available clinicopathological data, including recurrence-free survival following BCG therapy. 

## 2. Results

### 2.1. TERTp and FGFR3 Mutation Analysis

In the 125 BCG-treated NMIBC (BCG-NMIBC) tumors screened for *TERTp* mutations, 56.0% (70/125) of the cases were mutated. The c.1-124G>A mutation was detected in 36.8% (46/125) and the c.1-146G>A in 17.6% (22/125). In two cases (1.6%), both c.-124G>A and c.1-146 G>A mutations were observed (Table 1). *FGFR3* mutations (exons 7, 10 and 15) were evaluated in 107 cases. In the tumors screened for *FGFR3* mutations, 44.9% (48/107) of the cases were mutated (Table 1). The large majority was mutated in exon 7 and less frequently in exons 10 and 15 (Table 1). When analyzing *FGFR3* cases with only mutations in exon 7 (45 cases), 42.2% (19/45) presented the p.R248C mutation whereas the p.S249C mutation was present in 55.6% (25/45). One case harbored both mutations (2.2%). A comparison between *TERTp* mutation status and *FGFR3* status revealed no significant association between the two genetic events in the BCG-NMI tumors. The rs2853669 SNP was evaluated in 98 cases; rs2853669 AA genotype accounted for 39.8% (39/98) of the cases, AG genotype for 48.0% (47/98) and GG genotype for 12.2% (12/98) (Table 1).

### 2.2. Clinicopathological Characteristics and Genetic Alterations

A comparison between the clinicopathological characteristics of *TERTp* wild type and mutated cases was performed (Table 2). An association was found between *TERTp* mutations and recurrence status prior to BCG therapy, where an over-representation of *TERTp* mutations in primary tumors when compared with recurrent tumors can be detected (61.4% vs. 38.6%, *p* = 0.048).

In the BCG-NMIBC cases a statistically significant association between tumor size and *FGFR3* p.R248C mutations was found (*p* = 0.048). There was an over-representation of the mutation presence among tumors larger than 3 cm in comparison with the smaller ones (27.9% vs. 11.5%), Table S2. However, multivariate analysis revealed that FGFR3 p.R248C is not independently associated with tumor size.

The stratification of tumors in two groups, those wild type for both *TERTp* and *FGFR3* and those mutated for any, did not present statistically significant differences in the clinicopathological characteristics. Regarding the relationship of the studied polymorphism and clinicopathological features, an over-representation of the rs2853669 AA genotype was found in high-grade tumors when compared with low-grade tumors (77.4% vs. 22.6%, *p* = 0.018) (Table 3). 

### 2.3. Clinicopathological and Molecular Characteristics with BCG Therapy Success

Prior to tumor sampling, the BCG-NMIBC patients were treated with a scheme of BCG intravesical therapy. We evaluated how the clinicopathological characteristics affected BCG therapy outcome. Success was defined as no recurrence detected until the last surveillance check-up. Failure was defined as any recurrence after BCG treatment. After a univariate analysis, the age group ≥65 years (hazard ratio (HR): 2.827; 95% CI: 1.481–5.398; *p* = 0.002), multifocality (HR: 2.000; 95% CI: 1.096-3.649; *p =* 0.024) and maintenance BCG (mBCG) schedule (HR: 0.505; 95% CI: 0.282-0.902; *p* = 0.021) were the only variables significantly associated with the outcome, Table S1.

Next, we evaluated if the molecular characteristics have an effect on BCG therapy success. We performed a univariate analysis considering *TERTp* and *FGFR3* mutations and BCG therapy success. No statistically significant association was found on univariate analysis. To adjust for the effect of age group, multifocality, and BCG schedule on treatment success, we then performed a multivariate Cox regression analysis. When adjusted, the effect of status (wild type vs. mutated) for *TERTp* and *FGFR3* remained nonsignificant (Table 4 and Table 5). However, when we considered *TERTp* c.1-146G>A carriers against *TERTp* non c.1-146G>A carriers (either *TERTp* wild type or c.1-124G>A), the c.1-146G>A mutation was significantly associated with therapy success (HR: 0.382; 95% CI: 0.150-0.971; *p* = 0.043) (Table 4 and Table 5). In our series, the *TERTp* mutation c.1-146G>A was an independent predictor of therapy sucess following BCG intravesical therapy.

We further investigated the possible role of *TERTp* genetic events in predicting BCG therapy success by evaluating the presence of the single nucleotide polymorphism rs2853669 in the BCG-NMIBC series. We characterized cases as either carrier or noncarrier. No significant association was found for rs2853669 *per se*, or for *TERTp* mutation effect after splitting for rs2853669. 

### 2.4. TERTp Mutations and Recurrence-Free Survival

**Figure 1:**
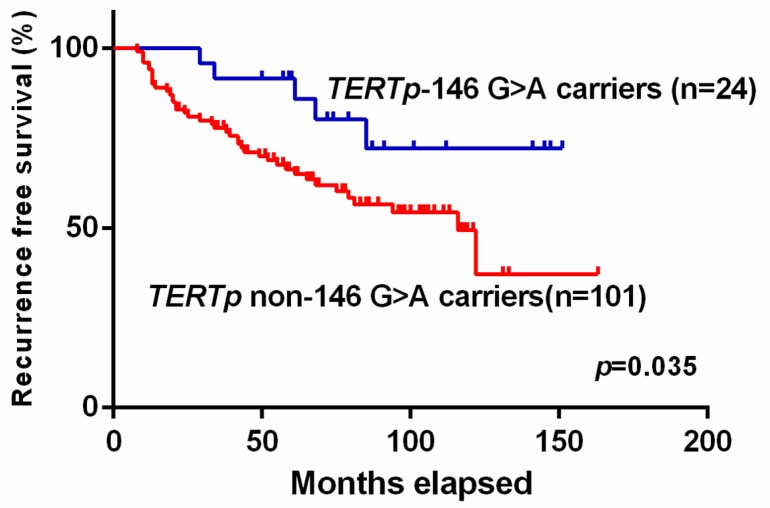
Kaplan–Meier recurrence-free survival function of BCG-NMIBC patients, grouped according to *TERTp* c.1-146G>A carriers against *TERTp* non c.1-146G>A carriers (either *TERTp* wild type or c.1-124G>A). Overall comparison of recurrence-free survival rates was performed using the log-rank test.

The recurrence-free survival function of all 125 BCG-treated NMIBC patients, grouped according to the existence of a *TERTp* mutation, was evaluated and log-rank testing revealed no statistically significant difference for either group (Figure S1). When considering *TERTp* c.1-146G>A carriers against *TERTp* non c.1-146G>A carriers (either *TERTp* wild type or c.1-124G>A), the *TERTp c.1*-146G>A patients presented a longer recurrence-free survival in comparison with the noncarriers (mean 126 months vs. mean 100 months, log rank *p* = 0.035) (Figure 1).

## 3. Discussion

*TERTp* mutations were reported in 52% to 85% of bladder cancer (BC) cases, depending on the series [10,13,14,16,17,18,19]. These results rank *TERTp* mutations as one of the most common genomic events observed in BC and possibly as the most frequent. Of all the *TERTp* mutations, c.1-124G>A has been consistently reported as the most frequent, detected in 88% to 95% of the positive cases [10,13,14,16,17,18,19]. In this study composed of BCG-treated NMIBC tumors, we report an overall *TERTp* mutation prevalence of 56.0%, in accordance with previously reported studies [10,13,14,16,17,18,19]. Conflicting results have been reported on the association between *TERTp* mutations and clinical stage and/or grade of bladder tumors. Wu et al. [19] found that *TERTp* mutations were more prevalent in MI tumors than in NMI tumors and in patients with advanced tumor stages. On the other hand, other studies reported no association between mutation status and stage or grade [13,14]. The results we present here support that *TERTp* mutations rates are not significantly different across grades or stages in this subset of BCG-treated NMIBC. However, it should be taken into account that this subset represents a group of particularly aggressive NMI tumors (BCG-NMIBC), and comparisons with NMIBC subseries in other studies must be made with caution. 

Found in approximately 70% of tumors, *FGFR3* activating mutations are regarded as important genetic events in the NMI phenotype [4]. We found that 44.9% of the BCG-NMIBC cases were mutated for *FGFR3*. As *FGFR3* mutations are associated with low-grade and low-stage tumors and seem to predict a more favorable clinical outcome among patients with NMI tumors [3,25], it was expectable that the more aggressive BCG-NMIBC tumors presented lower mutation rates than those reported in other series [4]. Analyzing the specific *FGFR3* mutation distribution, a novel pattern emerges; previously, in NMIBC series, the most frequent mutations were at the exon 7 p.S249C (66.6% overall, 87.3% of the exon 7 mutations) and p.R248C (9.7% overall, 12.0% of the exon 7 mutations), and mutations in exon 10 and 15 were infrequent [26]. The *FGFR3* mutations detected in this study were mostly present on exon 7 (91.7%), but we observed a different prevalence of the specific mutations, with p.S249C accounting for 52.0%, and an enrichment for p.R248C, with 39.6%. In the BCG-NMIBC cases wild type and mutated for FGFR3, a statistically significant association between tumor size and FGFR3 p.R248C mutation was found (*p* = 0.048). However, multivariate analysis revealed that FGFR3 p.R248C is not independently associated with tumor size. One can discuss if FGFR3 p.R248C mutations are associated with more aggressive tumors, since it is particularly enriched in this subset of tumors and associated with larger tumor size in a univariate analysis. Loss of association in multivariate analysis may indicate that this association may be due to the influence of other clinicopathological features or, more likely, the analyzed cohort is too low to robustly perform this analysis. Further studies are required to elucidate these assumptions and findings. Finally, no significant association was found between the presence of *TERTp* and *FGFR3* mutations.

Intravesical BCG therapy is used as prophylaxis against NMIBC recurrences after tumor resection and it is in fact regarded as one of the first and most successful of all oncological immunotherapies [27,28]. BCG intravesical instillation results in multiple immune reactions. Although the precise immunological mechanism of BCG therapy is not clear, it appears to act through three main actions—infection of urothelial cells or bladder cancer cells, induction of immune responses, and induction of anti-tumour effects [27]. Although effective, 30% to 40% of the cases still show either intolerance or recurrence after BCG treatment, demanding life-long follow-up and repeated courses of treatment [24]. This results in extreme discomfort for the patients and exceedingly high financial costs, and ranks BC as the most expensive cancer per patient [3]. Biomarkers that could help identify which patients were more likely to respond to BCG versus those with risk of recurrence — those who would benefit the most from either a tighter surveillance or a different treatment — would be very useful in optimizing the clinical care offered to BC patients. In this study, we report the effects of *TERTp* and *FGFR3* mutations in BCG therapy success (recurrence or nonrecurrence) and recurrence-free survival. Age at BCG treatment, multifocality, and BCG schedule were independent predictors of BCG therapy success (defined as no recurrence), with the age group ≥65 years and multifocal tumours associated with a higher risk of recurrence, whereas mBCG schedule was associated with a lower risk of recurrence. These results are concordant with previous reports [29]. After adjusting for age, multifocality, and BCG schedule, we found no association between *FGFR3* mutations and BGC therapy success. Similarly, *TERTp*-mutated cases as a whole showed no difference when compared to wild type cases. However, when we compared carriers of the *TERTp* c.1-146G > A mutation against those without this mutation, we observed that this specific mutation was an independent predictor of better outcome (delayed or nonrecurrence). 

Recently, Rachakonda et al. reported that a common polymorphism within a pre-existing Ets2 binding site in *TERTp*, rs2853669, acts as a modifier of the mutations’ effect on survival and tumor recurrence [18]. The patients with the *TERTp* mutations presented a poorer survival in the absence, but not in the presence of the variant allele (G) of the polymorphism [18]. TERTp mutations in the absence of the variant allele were highly associated with disease recurrence in patients with Tis, Ta, and T1 tumors [18]. To further investigate this, we screened BCG-NMIBC tumors for this common SNP. We found that rs2853669 carrier status did not modulate *TERTp* effect on BCG therapy success in our series, however, there was an association of rs2853669 AA carriers with tumors of higher grade. This association is in accordance with what Rachakonda and others described. In patients that harbor the germline rs2853669 AA genotype, the TERTp mutation effect is not reverted in the BC tumor. As Rachakonda described, patients with this combination (germline rs2853669 G absence, TERTp tumor positive) present poorer survival and increased disease recurrence, which are features compatible with the presence of more aggressive tumors, such as higher grade.

Finally, we analyzed recurrence-free survival after BCG treatment, comparing *TERTp* and *FGFR3* mutation status and specific mutations and rs2853669 carrier status. Kaplan–Meier survival analysis showed a promising recurrence-free survival advantage for those c.1-146G>A mutation carriers. Our results demonstrate that BCG-NMIBC c.1-146G>A mutation carriers are three time less likely to recur after BCG therapy and may have more favorable recurrence-free survival rates when compared to both *TERTp* wild type and c.1-124G>A cases. To interpret these findings, it is important to note that what we are evaluating is how *TERTp* mutations modulate the tumor response after BCG therapy. It has previously been suggested that the mechanism of BCG therapeutic effects on BC is related to its ability to reduce telomerase (*TERT*) activity [30] — we may speculate that c.1-146G>A mutated tumors might be more susceptible to the reduction of telomerase activity by BCG. As reported by Huang et al., *TERTp* mutations are associated with higher *TERT* transcription levels compared to wild type promoters but *TERTp* c.1-146G>A carriers have lower transcriptional capacity than those with the c.1-124G>A mutation [7]. We can speculate that the higher *TERT* expression induced by the c.1-124G>A mutation partially impairs BCG capability to sufficiently reduce telomeric activity to a therapeutic level — a level that could be achieved in a c.1-146G>A setting. Also, the lower frequency of *TERTp* mutations in BCG-treated recurrent tumors when compared to primary tumors could be explained by the enhanced BCG action on tumor cells harboring *TERTp* mutations, leading to clonal selection pressure towards cells harboring other alterations (such as *FGFR3* mutations) in recurrent tumors, hence shifting the prevalence of recurrent tumors towards TERTp-negative tumors. More studies comparing *TERT* expression and telomerase activity before and after BCG therapy with the different *TERTp* mutations are required to further interpret our results. To our knowledge, this study is one of the first studies addressing *TERTp* and *FGFR3* mutations in a BCG-NMI series of BC patients. We found no association between *TERTp* mutations, as a whole, and tumor grade or stage. However, we observed that the specific *TERTp* c.1-146G>A mutation was an independent predictor of nonrecurrence after BCG therapy in the BCG-NMI tumors. Our results suggest that it might be relevant to further study the role of *TERTp* mutations in tumor recurrence and as predictive markers of response to BCG therapy.

## 4. Materials and Methods

### 4.1. Human Cancer Samples and Clinicopathological Data

Formalin-fixed, paraffin-embedded (FFPE) tissues were obtained from 125 patients with NMI bladder urothelial cell carcinoma treated with intravesical BCG therapy, with samples being collected at the time of transurethral resection before any BCG therapy administration. Patients underwent resection of the tumors in the Portuguese Institute of Oncology — Francisco Gentil (IPO) Porto. Hematoxylin-eosin-stained sections were reviewed according to the standard histopathological examination by two independent pathologists. Staging and grading were conducted according to the American Joint Committee on Cancer [31], and the 2004 WHO classification system [32]. Clinicopathological and follow-up data were retrieved from the files of IPO databases. Age refers to age at BCG treatment initiation in the BCG-NMIBC group. Recurrence status characterizes the BCG-NMIBC cases as either a primary newly diagnosed tumor selected for BCG therapy or, alternatively, as a recurrence of a previously resected NMI tumor (that did not fill the criteria for being included in the BCG-NMIBC group before) that is only now selected for BCG therapy. BCG therapy selection was performed according to the EORTC criteria previously described [22]. BCG schedule characterizes the treatment regimen used as maintenance (mBCG) or induction-only (iBCG) intravesical BCG instillation. BCG therapy success was defined as no recurrence and failure was defined as any recurrence after BCG treatment. Analysis of patients’ age by age groups (<65 years and ≥65 years) was recognized as an informative analysis and has been used previously [33]. All the procedures described in this study were in accordance with national and institutional ethical standards and previously approved by Local Ethical Review Committees (Ethics Committee of the Portuguese Institute of Oncology of Porto with the number CES IPOPFG-EPE 586/08 in 25 of September of 2008). According to Portuguese law, informed consent is not required for retrospective studies.

### 4.2. DNA Extraction, PCR, and Sanger Sequencing

DNA was obtained from FFPE (10-micron sections) after careful microdissection. DNA extraction was performed using an Ultraprep Tissue DNA Kit (AHN Biotechnologie, Nordhaussen, Germany) following manufacturer’s instructions. 

To screen for *TERTp* mutations, we analyzed by PCR followed by Sanger sequencing of the hotspots previously identified [10]. *TERTp* mutation analysis was performed with the pair of primers Fw *TERT*—5′-CAGCGCTGCCTGAAACTC-3′ and Rv *TERT*—5′-GTCCTGCCCCTTCACCTT-3′. Amplification of genomic DNA (25–100ng) was performed by PCR using the Qiagen Multiplex PCR kit (Qiagen, Hilden, Germany) according to the manufacturer’s instructions. Sequencing reaction was performed with the ABI Prism BigDye Terminator Kit (Perkin Elmer, Foster City, CA, USA), and the fragments were run in an ABI prism 3100 Genetic Analyzer (Perkin-Elmer). The sequencing reaction was performed in a forward direction, and an independent PCR amplification/sequencing, both in a forward and reverse direction, was performed in positive samples or samples that were inconclusive. To screen for *FGFR3,* we analyzed the hotspots previously identified in exon 7, 10, and 15 in 107 BCG-NMIBC cases (18 cases have been excluded due to insufficient DNA for the analysis) by PCR followed by Sanger sequencing. *FGFR3* exon 7, 10, and 15 mutation analysis was performed with the respective pairs of primers Fw Exon 7—5′-AGTGGCGGTGGTGGTGAGGGAG-3′ and Rv Exon 7—5′-GCACCGCCGTCTGGTTGG-3′; Fw Exon 10—5′-CAACGCCCATGTCTTTGCAG-3′ and Rv Exon 10—5′-AGGCGGCAGAGCGTCACAG-3′; Fw Exon 15—5′-GACCGAGGACAACGTGATG-3′ and Rv Exon 15—5′-GTGTGGGAAGGCGGTGTTG-3′. Subsequent steps followed the same methodology as outlined for the *TERT* promoter mutation screening. 

### 4.3. Single Nucleotide Polymorphism Assay

Screening for the rs2853669 polymorphism was performed in 98 BCG-NMIBC cases (27 cases were excluded due to insufficient DNA for the analysis) using the rs2853669 TaqMan^®^ SNP Genotyping Assay (Applied Biosystems, Foster City, USA). Peripheral blood DNA was extracted using a genomic DNA extraction kit (Qiagen). The purified genomic DNA was used for the assay. The procedure was performed according to manufacturer’s instructions.

### 4.4. Uromonitor Real-Time PCR screening Assay

Screening of 125 nonmuscle invasive BC tumors treated with BCG therapy (BCG-NMIBC) for *TERTp* mutations and *FGFR3* hotspot mutations were confirmed by using a specific IVD commercial kit Uromonitor^®^—Real-Time PCR kit for the amplification and detection of *TERTp* and *FGFR3* hotspot mutations (U-Monitor, Porto, Portugal), according to manufacturer’s instructions. 

### 4.5. Statistical Analysis

The statistical analysis was performed using IBM SPSS statistics software version 25.0. For the analysis of the relationship between patients’ age, we used the independent-samples *t*-test. Pearson’s Chi-square and Fisher’s exact test were used in the statistical analysis of the other parameters, according to sample size. Cox proportional hazard ratios were estimated to obtain risks of recurrence for cases in each molecular factor stratum before and after adjusting for other confounding variables. Kaplan–Meier survival curves were computed by each category of the potential prognostic factors and the log-rank and Breslow tests were applied to compare curves. Means were used instead of medians because some survival curves did not fall under 50%. Results were considered statistically significant if *p* < 0.05.

## Figures and Tables

**Table 1:** Telomerase reverse transcriptase gene promoter (*TERTp*) mutations, Fibroblast Growth Factor Receptor 3 (*FGFR3*) mutations, and rs2853669 prevalence across BCG-treated cases of nonmuscle invasive bladder cancers (BCG-NMIBC).

	BCG-NMIBC, *n* (%)
***TERTp***	
Wild type	55 (44.0)
Mutated	70 (56.0)
***Specific mutations***	
*c.1-124G>A*	46 (36.8)
*c.1-146G>A*	22 (17.6)
*c.1-124G>A/c.1-146G>A*	2 (1.6)
***FGFR3***	
Wild type	59 (55.1)
Mutated	48 (44.9)
***Specific mutations***	
*Exon 7 p.R248C*	19 (39.6)
*Exon 7 p.S249C*	25 (52.0)
*Exon 10 p.Y375C*	1 (2.1)
*Exon 7 p.R248C + p.S249C*	1(2.1)
*Exon 7 p.R248C + Exon 10 p.Y375C*	1(2.1)
*Exon 7 p.R248C + Exon 15 p.K652E*	1(2.1)
**rs2853669**	
AA	39 (39.8)
AG	47 (48.0)
GG	12 (12.2)

**Table 2:** Relation between clinicopathological data and *TERTp* mutation status in BCG-NMIBC.

	*TERTp*		
	Wild Type, *n* (%)	Mutated, *n* (%)	*p*-value
Age group			
<65 years	21 (38.2)	33 (47.1)	0.315
≥65 years	34 (61.8)	37 (52.9)	
Gender			
Female	11 (20.0)	8 (11.4)	0.185
Male	44 (80.0)	62 (88.6)	
Stage			
Ta	23 (41.8)	28 (40.0)	0.837
T1	32 (58.2)	42 (60.0)	
Grade			
Low	15 (27.3)	25 (35.7)	0.315
High	40 (72.7)	45 (64.3)	
Tumour size			
<3 cm	34 (63.0)	41 (58.6)	0.620
≥3 cm	20 (37.0)	29 (41.4)	
Multifocality			
No	28 (50.9)	32 (45.7)	0.564
Yes	27 (49.1)	38 (54.3)	
Recurrence status			
Primary	24 (43.6)	43 (61.4)	**0.048**
Recurrent	31 (56.4)	27 (38.6)	

*p*-Values obtained from Pearson’s Chi-Square test for gender, stage, grade, tumor size, and multifocality and recurrence, bold values indicate *p* < 0.05.

**Table 3:** Relation between clinicopathological data and *rs2853669* single nucleotide polymorphism (SNP) status in BCG-NMIBC.

	*rs2853669*		
	AA, *n* (%)	G Carrier, *n* (%)	*p*-value
Age group			
<65 years	17 (41.5)	22 (38.6)	0.775
≥65 years	24 (58.5)	35 (61.4)	
Gender			
Female	5 (37.5)	34 (40.5)	0.736
Male	9 (64.3)	50 (59.5)	
Stage			
Ta	15 (39.5)	24 (40.0)	0.959
T1	23 (60.5)	36 (60.0)	
Grade			
Low	7 (22.6)	32 (47.8)	**0.018**
High	24 (77.4)	35 (52.2)	
Tumour size			
<3 cm	22 (34.9)	17 (50.0)	0.148
≥3 cm	41 (65.1)	17 (50.0)	
Multifocality			
No	19 (43.2)	20 (37.0)	0.536
Yes	25 (56.8)	34 (63.0)	
Recurrence status			
Primary	22 (43.1)	17 (36.2)	0.481
Recurrent	29 (56.9)	30 (63.8)	

*p*-Values obtained from Pearson’s Chi-Square test for gender, stage, grade, tumor size, and multifocality and recurrence, bold values indicate *p* < 0.05.

**Table 4:** Univariate analysis of the relation between *TERTp* and *FGFR3* mutations and recurrence after BCG treatment.

	BCG Therapy			
	Success, *n* (%)	Failure, *n* (%)	HR (95% CI)	*p*-value
*TERTp*				
Wild type	34 (43.0)	21 (45.7)	1.0	0.580
Mutated	45 (57.0)	25 (54.3)	0.848 (0.473–1.520)	
*TERTp* genotype				
Wild type	34 (43.0)	21 (45.6)	1.0	
c.1-124G > A	26 (32.9)	20 (43.5)	1.158 (0.626–2.143)	0.639
c.1-146G > A	17 (21.5)	5 (10.9)	0.410 (0.152–1.108)	0.079
c.1-124G>A/c.1-146G>A	2 (2.5)	0 (0.0)	0.464 (0.040–5.327)	0.464
*TERTp* c.1-146G>A status				
c.1-146G>A carriers	60 (75.9)	41 (89.1)	1.0	**0.043**
non c.1-146G>A carriers	19 (24.1)	5 (10.9)	0.382 (0.150–0.971)	
*FGFR3*				
Wild type Mutated	39 (60.0) 26 (40.0)	20 (51.3) 19 (48.7)	1.0 1.336 (0.712–2.507)	0.367
*FGFR3* status				
Wild type	39 (60.0)	20 (51.3)	1.0	
p.R248C	12 (18.5)	7 (17.9)	1.158 (0.524–3.015)	0.608
p.S249C	14 (21.5)	11 (28.2)	0.410 (0.650–2.842)	0.415
p.R248C/p.S249C	0 (0.0)	1 (2.6)	1.584 (0.804–3.120)	0.184

*p*-values obtained from Wald test; bold values indicate *p* < 0.05. HR, Hazard Ratio; CI, Confidence Interval.

**Table 5:** Multivariate analysis and risk estimation of TERT c.1-146G>A mutation influence on BCG therapy outcome.

*TERTp c.1*-146G>A Status	HR ^a^	95% CI	*p*-value
c.1-146G>A carriers	1.0	Referent	
non c.1-146G>A carriers	0.256	0.098-0.667	0.005
Age ≥ 65 years	2.370	1.206-4.661	0.012
Multifocality	1.883	0.964-3.677	0.064
Recurrent tumor	1.352	0.703-2.600	0.367
iBCG schedule	2.225	1.211-4.088	0.010

HR, Hazard Ratio; CI, Confidence Interval. ^a^ adjusted for age, multifocality, recurrence status, and BCG schedule.

## Supplementary Materials

Supplementary Materials can be found at https://www.mdpi.com/1422-0067/21/3/947/s1.

## Abbreviations

BC	Bladder cancer
BCG	Bacillus Calmette–Guérin
BCG-NMI	BCG-treated nonmuscle invasive
NMI	Nonmuscle invasive
