# Supplementary material for: TERT Promoter Mutation as a Potential Predictive Biomarker in BCG-Treated Bladder Cancer Patients
Source: Int J Mol Sci. 2020 Jan 31;21(3):947. doi: 10.3390/ijms21030947 (PMC7037401; doi:10.3390/ijms21030947)
Supplement: Supplementary file 1 [file ijms-21-00947-s001.pdf]

TERT Promoter Mutation as a Potential Predictive Biomarker in BCG-Treated Bladder Cancer Patients

Rui Batista [1,2,3,†], Luís Lima [4,†], João Vinagre [1,2,3,†], Vasco Pinto [2,3], Joana Lyra [2,3], Valdemar Máximo [1,2,3], Lúcio Santos [4] and Paula Soares [1,2,3,*]

[1] Instituto de Investigação e Inovação em Saúde (i3S), 4200-135 Porto, Portugal; rbatista@ipatimup.pt (R.B.); jvinagre@ipatimup.pt (J.V.); vmaximo@ipatimup.pt (V.M.)
[2] Instituto de Patologia e Imunologia Molecular da Universidade do Porto (IPATIMUP), 4200-135 Porto, Portugal; vasco.sa.pinto@gmail.com (V.P.); joanaritalyra@gmail.com (J.L.)
[3] Department of xxxx, Faculdade de Medicina da Universidade do Porto (FMUP), 4200-319 Porto, Portugal;
[4] Grupo de Patologia e Terapêutica Experimental, Instituto Português de Oncologia do Porto FG, EPE (IPO-Porto), 4200-072 Porto, Portugal; luis14lima@gmail.com (L.L.); llarasantos@gmail.com (L.S.)
* Correspondence: psoares@ipatimup.pt; Tel.: +351-2255-70700
† These authors contributed equally to this work.

Received: 28 December 2019; Accepted: 30 January 2020; Published: date

Supplementary data

Supplementary Table S1. Relation between clinicopathological characteristics and recurrence after BCG treatment. *p* values obtained from Wald test; bold values indicate $p < 0.05$. HR, Hazard Ratio; CI, Confidence Interval

	total	BCG therapy		HR (95% CI)	*p*-value
	n (%)	success *n* (%)	failure *n* (%)		
Age group					
< 65 years	54 (43.2)	41 (51.9)	13 (28.3)	1.0	**0.002**
≥ 65 years	71 (56.8)	38 (48.1)	33 (71.7)	2.827 (1.481-5.398)	
Gender					
Female	19 (15.2)	15 (19.0)	4 (8.7)	1.0	0.179
Male	106 (84.8)	64 (81.0)	42 (91.3)	2.023 (0.724-5.654)	
Stage					
Ta	51 (40.8)	30 (38.0)	21 (45.7)	1.0	0.535
T1	74 (59.2)	49 (62.0)	25 (54.3)	1.202 (0.672-2.148)	
Grade					
Low	40 (32.0)	25 (31.6)	15 (32.6)	1.0	0.557
High	85 (68.0)	54 (68.4)	31 (67.4)	1.204 (0.648-2.238)	
Tumour size					
< 3 cm	75 (60.5)	46 (58.2)	29 (64.4)	1.0	0.541
≥ 3 cm	49 (39.5)	33 (41.8)	16 (35.6)	0.826 (0.448-1.524)	
Multifocality					
No	60 (48.0)	43 (54.4)	17 (37.0)	1.0	**0.024**
Yes	65 (52.0)	36 (45.6)	29 (63.0)	2.000 (1.096-3.649)	
Recurrence status					
Primary	67 (53.6)	48 (60.8)	19 (41.3)	1.0	0.051
Recurrent	58 (46.4)	31 (39.2)	27 (58.7)	1.795 (0.998-3.229)	
BCG schedule					
iBCG	48 (38.4)	24 (30.4)	24 (52.2)	1.0	**0.021**
mBCG	77 (61.6)	55 (69.6)	22 (47.8)	0.505 (0.282-0.902)	

Supplementary Table S2A. Relation between tumour size and mutation status of *FGFR3*. *p*-Values obtained from Pearson's Chi-Square test

	**Tumour size**		
	**< 3 cm**	**> 3 cm**	*p*-value
FGFR3 exon 7			

Wild type, *n* (%)	37 (60.7)	22 (51.2)	0.336
Mutated, *n* (%)	24 (39.3)	21 (48.8)	
genotype			
R248C	7 (11.5)	12 (27.9)	**0.048**
S249C	17 (27.9)	8 (18.6)	0.644
R248C + S249C	0 (0.0)	1(2.3)	-

Supplementary Table S2B. Multivariate analysis exploring the relation between tumour size and mutation status of FGFR3 adjusted to potential confounders. *p*-Values obtained from binary logistic regression test.

	**OR**	**95% CI**	***p*-value**
FGFR3 exon 7 *R248C*	0.373	0.116-1.202	0.099
Age≥ 65 years	1.414	0.505-3.963	0.510
T1 tumors	0.588	0.197-1.756	0.342
Multifocality	0.996	0.339-2.929	0.994
Recurrent tumor	3.037	0.977-9.443	0.055

OR: Odds Ratio; CI: Confidence Interval.

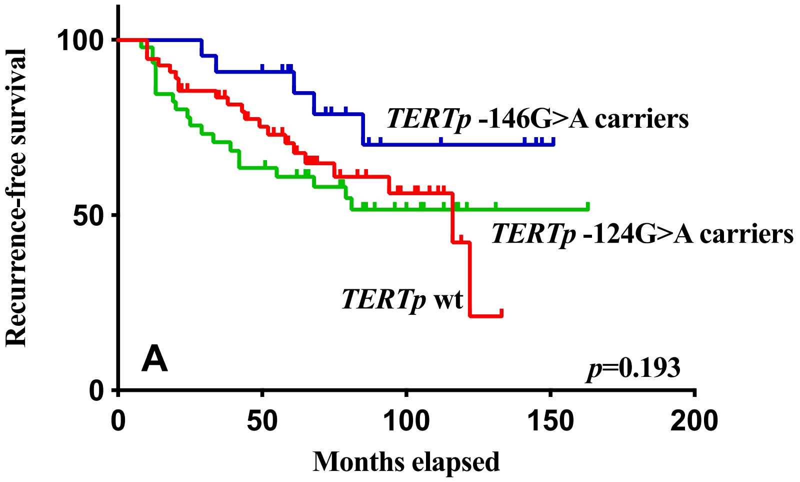

Supplementary Figure S1. Kaplan–Meier recurrence-free survival function of BCG-NMIBC patients, grouped according to *TERTp* as wild type, -124G > A, and -146G > A. Overall comparison of recurrence-free survival rates was performed using the log-rank test.
